# Abnormal Gas Diffusing Capacity and Portosystemic Shunt in Patients With Chronic Liver Disease

**DOI:** 10.4021/gr475e

**Published:** 2012-09-20

**Authors:** Moon-Seung Park, Min-Ho Lee, Yoo-Sin Park, Shin-Hee Kim, Min-Jung Kwak, Ju-Seop Kang

**Affiliations:** a Department of Internal Medicine, College of Medicine, Hanyang University, Seoul 133-791, South Korea; b Department of Pharmacology and Clinical Pharmacology Laboratory, Hanyang University, Seoul 133-791, South Korea; c Department of Informative Statistics, College of Natural Science, Pyongteak University, Pyonkteak 450-701, South Korea

**Keywords:** Pulmonary diffusion capacity, Portosystemic shunt (H/L ratio), DLco, DLco/VA, CAH, Child-Pugh class

## Abstract

**Background:**

Pulmonary dysfunctions including the hepatopulmonary syndrome and portosystemic shunt are important complications of hepatic cirrhosis. To investigate the severity and nature of abnormal gas diffusing capacity and its correlation to portosystemic shunt in patients with chronic liver disease.

**Methods:**

Forty-four patients with chronic liver disease (15 chronic active hepatitis (CAH), 16 Child-Pugh class A, and 13 Child-Pugh class B) without other diseases history were enrolled in the study. Evaluation of liver function tests, arterial blood gases analysis, ultrasonography, pulmonary function test including lung diffusing capacity of carbon monoxide (DLco), forced vital capacity(FVC), forced expiratory volume 1 seconds(FEV1), total lung capacity(TLC), DLco/AV(alveolar volume) and thallium-201 per rectum scintigraphy were performed. We were analyzed correlations between pulmonary function abnormalities and heart/liver (H/L) ratio in patients with chronic liver diseases.

**Results:**

In CAH, percentage of patients with DLco and DLco/VA (< 80%) was 22.2 % but it was significantly increased to 47.2-54.5% in Child-Pugh class A and B patients. The means of DLco and DLco/VA were significantly (P < 0.05) decreased in Child-Pugh class. The mean H/L ratio in Child-Pugh class B increased markedly (P < 0.01) than those with CAH and Child-Pugh class A. The frequency of specific pulmonary function abnormality in patients with Child-Pugh class B was significantly (P < 0.01) greater than those with Child-Pugh class A and CAH. There was a inverse linear correlation between H/L ratio and DLco (r = -0.339, P < 0.05) and DLco/VA (r = -0.480, P < 0.01).

**Conclusion:**

A total of 62% of patients with advanced liver disease have abnormal pulmonary diffusion capacity with a reduced DLco or DLco/VA and abnormal portosystemic shunt (increased H/L ratio) is common hemodynamic abnormality. Therefore, inverse linear correlation between DLco or DLco/VA and H/L ratio may be an important factor in predicting pulmonary complication and meaningful diagnostic and prognostic parameters in patients with advanced chronic liver disease.

## Introduction

The common pulmonary complications of chronic liver disease are arterial oxygenation abnormalities resulting from right-left shunt and/or ventilation-perfusion mismatch. The mechanism is unknown but is thought to be due to increased hepatic production or decreased hepatic clearance of vasodilating endogenous substances, possibly involving nitric oxide [1].

The frequent occurrence and severity of decreased arterial oxygenation in patients with liver disease were important things to pertinent management of patients in clinical setting. Hepatopulmonary syndrome is defined as the triad of liver disease, pulmonary gas exchange abnormalities leading to hypoxemia (low oxygen levels in the arterial blood), and widespread pulmonary vascular dilatation [1, 2].

Hepatopulmonary syndrome is one of the grave complications of chronic liver disease that is characterized by microscopic intrapulmonary arteriovenous dilatations, an increased alveolo-arterial oxygen difference with both chronic and acute hepatic failure in the absence of other cardiopulmonary diseases [3-5]. Hepatopulmonary syndrome occurs in approximately 20% of patients with chronic liver disease or portal hypertension. While there has been few established concerning the pathogenesis of hepatopulmonary syndrome, it has been possible to show that arterial oxygenation defect may be components resulting from ventilation-perfusion mismatching, increased intrapulmonary shunt, and limitations of oxygen diffusion.

The degree of severity of the hepatopulmonary syndrome based on abnormalities in oxygenation is vital because severity influences survival and is useful in determining the timing and risks of liver transplantation [1]. The patients of advanced liver disease showed a pulmonary dysfunction and the most frequent finding was impairment in DLco [6].

Chronic liver disease is sometimes associated with an abnormal increase in pulmonary vascular resistance, resulting in portopulmonary hypertension that is common determinant of portal hypertension and portosystemic shunting [7]. However, it is not known the pattern of change of pulmonary diffusing capacity and relationship between pulmonary diffusing capacity and portosystemic shunt in patients with chronic liver diseases.

Therefore, the aims of the present study were to investigate the severity and nature of abnormal gas diffusing capacity and its correlation with portosystemic shunt in 44 patients with various severities of chronic liver disease.

## Methods

### Patients

Patients eligible for this study was recruited and participated after giving their informed and signed consent, in accordance with the Helsinki II Declaration and the study protocol approved by the institutional review board (IRB) for Medical Research of the Hanyang University Medical Center.

A total of 44 patients with chronic liver disease without ascites and any history of primary lung or heart disease that had admitted for liver biopsy at the Gastroenterology Center of the Hanyang University Medical Center were evaluated consecutively during 2 years. All patients underwent the record of medical history including smoking status and the physical examination including pitting edema in anterior tibia area and classified into chronic active hepatitis (CAH), Child-Pugh class A and B according to hepatic histopathology, 15 CAH patients were allocated to control group and Child-Pugh class A and B patients enrolled our study. Exclusion criteria are as follows: Child-Pugh class C, history of pulmonary or heart disease, uncontrolled ascites, vasculitis disease which affects pulmonary DLco. Child-Pugh classification and other clinical findings were confirmed by a hepatologist. All patients underwent measurements of serum albumin, serum total bilirubin, serum cholesterol, serum alanine aminotransferase(ALT), aspatate aminotransferase(AST), alkaline phosphatase, serum ν-globulin; prothrombin time, abdominal sonography for detection of ascites (Table 1).

**Table 1:** Demographic and Clinical Characteristics of the Patients

Variables	CAH	Class A	Class B		Total
Numbers of patients	15	16	13		44
Age (years, mean ± SD)	32.3 ± 8.5	47.2 ± 12.1	48.6 ± 10.3		40.3 ± 8.3
Gender (male/female)	9/6	12/4	9/4		30/14
Etiology (numbers (%))					
HBV induced	14 (93.3)	9 (56.3)	12 (92.3)		35 (79.5)
HCV induced	0 (0.0)	4 (25.0)	0 (0.0)		4 (9.1)
Alcohol-related	1 (6.7)	2 (12.5)	1 (7.7)		4 (9.1)
Unknown	0 (0.0)	1 (6.3)	0 (0.0)		1 (2.3)
Smoking status (numbers (%))					
No smoking	7 (46.7)	6 (37.5)	4 (30.7)	17 (38.6)	
Former or current smoker	8 (53.3)	10 (62.5)	9 (69.3)	27 (61.4)	
Evaluation of chest radiography (numbers (%))					
Normal	14 (93.3)	16(100.0)	12 (92.3)		42 (95.5)
Abnormal	1 (6.7)	0 (0.0)	1 (7.7)		2 (4.5)
Liver function test (Mean ± S.D)					
ALT (U/L)	116.9 ± 86.6	61.6 ± 36.2	51.3 ± 33.9*		
AST (U/L)	69.3 ± 50.1	62.6 ± 36.2	60.2 ± 24.5		
TB (mg/dL)	1.9 ± 0.7	1.1 ± 0.5	2.1 ± 0.8		
Albumin (g/dL)	4.4 ± 0.6	4.4 ± 0.6	3.5 ± 0.6		
PT (%)	95.8 ± 11.2	91.3 ± 12.9	64.9 ± 10.6*		

The numbers of each variable among the groups were analyzed by Chi-square analysis at P < 0.05 level. CAH: chronic acute hepatitis; Class A: Child-Pugh Class A; Class B: Child-Pugh Class B; HBV: hepatitis B virus; HCV: hepatitis C virus. Asterisk (*) shows significant differences among the three groups in each variable by ANOVA at P < 0.05 level. ALT: alanine aminotransferase; AST: aspartate aminotransferase; TB: total bilirubin; PT: prothrombin time.

### Measurement of portosystemic shunt (Heart/Liver ratio)

Thallium 201 per rectal scintigraphy test was performed in a blinded fashion in all patients according to the procedure described by Tonami et al [8] that is the heart/liver (H/L) uptake ratio at 20 min after thallium-201 administration [9].

### Pulmonary function test

Measurements of forced vital capacity (FVC), forced expiratory volume in first second (FEV1), carbon monoxide diffusion capacity (DLco) and ratio of DLco to alveolar ventilation (DLco/VA) were done in accordance with American Thoracic Society criteria by single breath technique [10, 11]. Results were recorded as the percentage of the predicted values [12, 13] and above 80% of predicted values were considered as normal. All DLco data were adjusted for hemoglobulin level. Subdivisions of lung volumes were determined by the helium dilution method and expressed as a percentage of the predicted value [14, 15]. Spirometric indices were calculated from the best three satisfactory breaths and compared with its predicted values [10]. Posteroanterior chest radiographs were obtained within 2 days of pulmonary function tests and interpreted by a radiologist who was unaware of the patient’s clinical and pathophysiological findings. Chest radiographs were analyzed for any abnormal finding such as change of lung volumes, thoracic deformity, plural effusion, abnormal increased interstitial lung marking and atelectasis etc.

### Evaluation of pulmonary functions and its correlation to disease category and portosystemic shunt

The differences between disease categories were analyzed with ANOVA. Subjects in each group were also categorized as non-smoker and experienced smokers to exclude the effect of smoking on lung function. The effect of smoking according to disease category was investigated using two-way ANOVA. Pearson correlation analysis was used to investigate the relationship between pulmonary function and heart/liver ratio.

## Results

### Demographic and clinical characteristics

All of the patients had undergone liver biopsies for initial diagnosis before this study: 15 patients with chronic active hepatitis (CAH), 16 patients with Child-Pugh class A and 13 patients with Child-Pugh class B. Demographical and clinical characteristics of the patients are shown in Table 1. The 44 patients consecutively selected had a mean age of 40.25 ± 8.29 years (17 - 67 years) and consisted of 30 males (68%) and 14 females (32%). The mean age of the patients with Child-Pugh class A and B were 14.9 - 16.3 years older than the patients with CAH. The major cause of chronic hepatitis was hepatitis B virus (HBV, 79.6%) and followed by hepatitis C virus (HCV, 9.1%), alcohol (9.1%), and unknown cause (2.3%) in tested patients. The proportions of nonsmokers in each category were 46.7%, 37.5% and 30.7% in CAH, Child class A and B, respectively. As the Table 1 was shown, ALT and AST levels were decreased significantly in Child-Pugh class A and B. Total bilirubin was increased significantly (P < 0.05) in Child-Pugh class B compared to CAH and Child-Pugh class A. Prothrombin time was significantly decreased in Child-Pugh class B (P < 0.05).

### H/L ratio of the patient with each disease category

**Figure 1:**
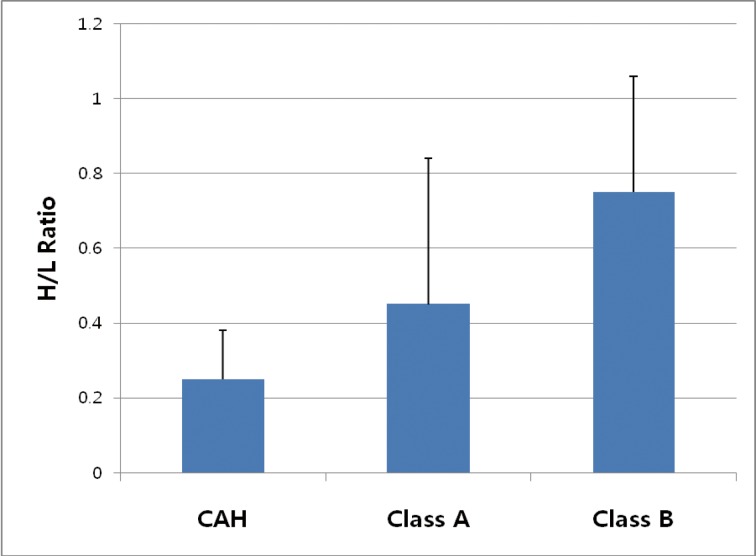
Mean values of H/L ratios of the patients. Asterisk (*) shows the significant difference among 3 groups by ANOVA (P < 0.01). CAH: chronic active hepatitis; Class A: Child-Pugh class A; Class B: Child-Pugh class B.

The H/L ratio in thallium-201 per rectum scintigraphy was used to measure amount of portosystemic shunt indirectly and it is useful in distinguishing CAH and cirrhosis and management of patients with cirrhosis [16]. In healthy subjects, Thallium-201 chloride absorbed from rectum passes through the portal system into liver that most of the radioactivity will be fixed by hepatocyte. In cirrhotic patients, a part of the absorbed radioactivity enters directly into the systemic circulation by portosystemic shunt producing a higher uptake in other organs including the myocardium. As shown in Figure 1, the H/L ratio in patients with Child-Pugh class B was significantly increased than those of patients with CAH and Child-Pugh class A in this study (P < 0.01).

### Pulmonary functions in the patients with each disease category

In the Table 1, two patients among tested showed increased lung volume in patients with CAH and mild pulmonary congestion in patients with Child-Pugh class B. The others showed no abnormal findings in chest radiograph. There was no severe hypoxemia in all groups, but was shown significant difference in proportions of DLco/VA that may be explained by alterations in gas exchanges [17, 18]. In CAH, percentage of patients with diffusion capacity for CO (DLco) and DLco/VA values lower than 80% of the predicted value was 22.2 % but it was significantly increased to 47.2-54.5% in patients with Child-Pugh class A and B and the means of DLco and DLco/VA were significantly decreased in patients with Child-Pugh class B than patients with CAH (P < 0.05) by Turkey’s multiple comparison test for the different sample sizes.

### Frequency of specific pulmonary abnormalities in patient with each disease category

The patients were classified as obstructive defect if the observed ratio of one-second forced expiratory volume (FEV1) to forced vital volume (FVC) was 2 or more standard deviations below the predicted FEV1/FVC ratio [19] and restrictive defect if the total lung capacity (TLC) was less than 80% of predicted [14]. Diffusion impairment was diagnosed if the DLco was less than 75% of the predicted value [12] after correction of the anemia [20], provided the DLco/VA was less than predicted value [12]. The proportion of the patients with abnormalities in specific pulmonary test is shown in Table 2. Among the patient, 48.3% of patients with chronic liver disease revealed pulmonary abnormality. The frequency of specific abnormality of pulmonary function in patients with Child-Pugh class B (52.9% of total frequency) was much greater than those of patients with Child-Pugh class A (29.4% of total frequency) and CAH (17.6% of total frequency), 62% of the patients with Child-Pugh class B and 25% of the patients with Child-Pugh class A showed diffusion impairment. In contrast to relatively high prevalence of diffusion impairment, restrictive defect was noted in < 10% of the total patients and no significant airflow obstructive defect on gas exchange was observed.

**Table 2:** Pulmonary Function and Arterial Blood Gas Analysis of the Patients

Variables	CAH (n = 15)	Class A (n = 16)	Class B (n = 13)
Spirometry and Single breath technique (mean % of predicted value ± S.D.)			
FVC	91.7 ± 7.4	103.2 ± 11.9	98.2 ± 11.8
FEV1	94.7 ± 8.9	98.0 ± 12.2	96.5 ± 10.4
FEV1/FVC	103.2 ± 4.9	94.9 ± 2.9	101.1 ± 7.8
TLC	91.9 ± 9.9	96.1 ± 10.8	94.9 ± 11.9
DLco	81.1 ± 11.9	77.7 ± 12.6*	69.3 ± 9.4*
DLco/VA	94.3 ± 9.6	85.4 ± 17.4*	79.1 ± 8.4*
Arterial blood gas analysis			
PaO_2_ (mmHg)	96.3 ± 8.4	88.7 ± 13.6	88.1 ± 10.8
SaO_2_ (%)	98.2 ± 1.4	95.5 ± 4.8	96.5 ± 1.2
Pulmonary function abnormality (case, %)			
Diffusion impairment††	1 (6.7)	4 (25.0)	8 (61.5)
Restrictive defect	2 (13.3)	1 (6.3)	1 (7.7)
Obstructive defect	0 (0.0)	0 (0.0)	0 (0.0)
Total case (%)†	3 (20.0)	5 (31.3)	9 (69.2)

* shows the significant differences among the three groups in each variable by ANOVA at P < 0.01 level. † and †† show the significant differences among the groups in each variable by Chi-square analysis at P < 0.05 and P < 0.01, respectively. FVC: forced vital capacity; FEV1: forced expiratory volume for 1 second; TLC: total lung capacity; DLco: diffusing capacity for carbon monoxide; VA: alveolar volume; PaO_2_: partial pressure of arterial oxygen; SaO_2_ : saturation of arterial oxygen.

### Correlation of pulmonary functions to disease category and smoking status

Two-way ANOVA analysis for disease category and smoking status was applied to each result of pulmonary function tests that revealed for disease category identified significance difference among pulmonary function results such as FVC, FEV1/FVC, DLco, and DLco/VA (P < 0.05) in the Table 2. There were no significant differences in mean values for any results of pulmonary function test and smoking status. But there was some interaction between disease category and smoking status in FVC (P = 0.072).

### Correlation between pulmonary functions and H/L uptake

Table 3 represents Pearson's correlation between the H/L ratio and each results of pulmonary function test. There was a significant inverse linear correlation between H/L ratio and DLco(r = -0.3395, P < 0.05) and DLco/VA (r = -0.4801, P < 0.001).

**Table 3:** Differences of Pulmonary Function According to Disease Category and Smoking Status and Correlation With H/L Ratio

Variables	FVC	FEV1	FEV1/FVC	TLC	DLco	DLco/VA
Disease Category	0.022*	0.679	0.012*	0.538	0.044*	0.013*
Smoking Status	0.100	0.190	0.541	0.716	0.522	0.886
Interaction	0.072	0.244	0.316	0.612	0.538	0.547
H/L ratio	0.2653	0.089	-0.3072†	0.1666	-0.3395†	-0.4801††

All the data were presented as p-values by two-way ANOVA, and * shows the significant p-value under 0.05 level. † and †† show the significant correlations between pulmonary function such as spirometric indices and H/L ratio by Pearson’s correlation analysis at P < 0.05 and P < 0.001 levels, respectively. Disease category includes ‘chronic active hepatitis’, ‘Child-Pugh class A’ and ‘Child-Pugh class B’. Smoking status includes ‘nonsmokers’ and ‘former or current smokers’. Interaction means relationship between disease category and smoking status.

## Discussion

Pulmonary function abnormalities were noted in chronic liver disease that is related both the degree of hepatocellular damage and to the hemodynamic changes induced by portosystemic shunt. One of the severe complications of chronic liver disease is hepatopulmonary syndrome that is defined as a triad of liver disease and/or portal hypertension, intrapulmonary vascular dilatation and hypoxemia, in the absence of detectable primary cardiopulmonary disease [5, 21].

To evaluate the pulmonary function abnormalities in the patients with different severity of chronic liver diseases such as CAH, Child-Pugh class A and B, we tried to analyzed prospectively liver and pulmonary function test. 62% of the patients with Child-Pugh class B and 25% of the patients with Child-Pugh class A showed diffusion impairment in 44 patients with chronic liver diseases. Among patients with CAH, only 6.7% of them showed reduced diffusion capacity. Based on the this finding, advanced hepatic dysfunction, with associated with hyperdynamic circulation, has been suggested as being the probable setting for the development of pulmonary diffusion impairment that is associated with pulmonary gas exchange abnormalities. Other specific pulmonary abnormalities and chest radiograph were unremarkable.

Even if the cause of the reduced diffusion capacity in the patients with advanced liver diseases such as Child-Pugh class A and B in this study is not clear, but one possible explanation is that the gas diffusion from the alveoli to the capillary is interfered because of pulmonary vascular dysfunction [3]. Mild hypoxemia is found in the apparent absence of cardiac and pulmonary disease in approximately one-third of patients with cirrhosis [22]. Mild arterial hypoxemia is due to ventilation-perfusion mismatch characterized by increased blood flow ahile alveolar ventilation is uniformly preserved, and in 30% of patients with cirrhosis, this blood flow is enhanced by the absence or impairment of hypoxic pulmonary vasoconstriction [23]. The severity of hypoxemia appears to be directly related to the extent of intrapulmonary shunt, diffusion-perfusion impairment or both, in contrast, the role of portopulmonary vascular communications is marginal [24, 25]. Impaired hypoxic vasoconstriction and increased pulmonary blood flow may contribute to ventilation-perfusion abnormalities [26, 27]. In the advanced cases, major intrapulmonary shunting and diffusion abnormalities may coexist as a result of the intrapulmonary vascular dilatation in which the increase in capillary diameter prevents oxygen molecules from diffusing to the center of the capillary to oxygenate the hemoglobin. This diffusion impairment is made worse by the high cardiac output, which reduces the capillary transit time and therefore the amount of time available for oxygen diffusion to occur [3]. Because intrapulmonary vascular dilatation in cirrhosis was evident in several reports using contrast-enhanced echocardiography has become the gold standard for diagnosis of intrapulmonary vascular dilatation [4, 5, 22, 28]. Another possible explanation in this defect is thickened alveolar capillary membrane such as fibrosing alveolitis [29]. Because most findings of chest radiograph and pulmonary function abnormalities were insignificant, we can be excluded the possibility of restrictive lung disease as a cause of diffusion defect in this study. Because mean of TLC between patients with CAH and Child-Pugh class A were not different in aspect of restrictive defect and FVC were also within normal limit in two disease categories, we could not find any significant meaning of decreased FVC in patients with CAH. In contrast to relatively high prevalence of diffusion impairment, prevalence of restrictive defect was noted in low (< 10%) of the patients with Child-Pugh class A and B, while clinically significant airflow obstruction that shown abnormal FEV1/FCV ratio was infrequent in all patients. There was no severe hypoxemia in all groups, but was shown significantly difference in proportions of patient with low DLco/VA (< 80 % of predicted values) are 12% in CAH and 47.2% in Child-Pugh class A and 54.5% in Child-Pugh class B that may be explained by alterations in gas exchanges [17]. Mean values of DLco and DLco/VA that is significantly decreased in Child-Pugh class A and B compared to CAH. These results indicate that the main pulmonary dysfunction regarding gas exchange is a diffusion-perfusion defect that may be explained by the difficulty in red blood cell oxygenation due to capillary dilatation and a high cardiac output in hepatopulmonary syndrome. There were no significant differences in mean values for any results of pulmonary function test between genders and smoking status.

The H/L ratio in thallium-201 per rectum scintigraphy was used to measure amount of portosystemic shunt indirectly and it is useful in distinguishing CAH and cirrhosis and management of cirrhotic patients [16]. In healthy subjects, Thallium-201 chloride absorbed from rectum passes through the portal system into liver that most of the radioactivity will be fixed in hepatocyte. In cirrhotic patients, a part of the absorbed radioactivity enters directly into the systemic circulation by portosystemic shunt producing a higher uptake in other organs including the myocardium. Therefore, the ratio of heart-liver radioactivities can be used as a quantitative index of portosystemic shunt. In the present study, it showed reverse correlation between portosystemic shunt and pulmonary diffusing capacity. The strongest relationships (those that are significant at 0.01) are the reverse correlation between the H/L ratio and DLco/VA and moderate relationships (those that are significant at 0.05) are the reverse correlation between the H/L ratio and DLco and FEV1/FVC. The results suggest that diffusion impairment is probably one of the results of the systemic vascular dilatation in chronic liver disease. In chronic liver disease, vascular dilatation occurs not only in pulmonary vessel but also in other vascular systems including the skin, kidney, brain and portal vein that are related to cutaneous spider angioma, functional renal and pulmonary dysfunction, portal hypertension, and brain edema [19]. Pathogenesis of hepatopulmonary syndrome is a decrease in diffusing capacity due to precapillary dilatation, arteriovenous shunt, and ventilation-perfusion mismatching that may be result in hypoxemia. In the situation of hepatopulmonary syndrome occurring in decompensated liver cirrhosis, a good therapeutic outcome cannot be expected [20]. In order to choose an optimal time for medical therapy and develop an efficient drug for patients with chronic liver disease, it is important to how when the vascular dilatation and arteriovenous shunt starts in the progression of chronic liver disease. To consolidate the evidence that diffusion impairment in chronic liver disease is worsen as the disease progresses, the investigators have plan to fallow up the patients enrolled in this study to further examine the pulmonary function test and thallium-201 per rectum scintigraphy. In conclusion, even if specific abnormalities in pulmonary function test and chest radiograph were insignificant, 61.4% of patients with Child-Pugh class B and 25% of patients with Child-Pugh class A showed diffusion impairment and showed significant relationship regarding the reverse correlation between degree of heart/liver shunting and pulmonary diffusing capacity depending on severity of liver disease. Therefore, diffusing capacity for CO (DLco) and DLco/VA values along with H/L ratio may be an important diagnostic marker for predicting occurrence of pulmonary complication in progressing chronic liver disease.
